# Effects of Mirror Neurons-Based Rehabilitation Techniques in Hand Injuries: A Systematic Review and Meta-Analysis

**DOI:** 10.3390/ijerph19095526

**Published:** 2022-05-02

**Authors:** Marco Tofani, Luigino Santecchia, Antonella Conte, Anna Berardi, Giovanni Galeoto, Carla Sogos, Maurizio Petrarca, Francescaroberta Panuccio, Enrico Castelli

**Affiliations:** 1 Professional Development, Continuous Education and Research Service, Bambino Gesù Children’s Hospital, 00165 Rome, Italy; 2 Department of Human Neurosciences, Sapienza University of Rome, 00185 Rome, Italy; antonella.conte@uniroma1.it (A.C.); anna.berardi@uniroma1.it (A.B.); giovanni.galeoto@uniroma1.it (G.G.); carla.sogos@uniroma1.it (C.S.); 3 Orthopedic Unit, Department of Surgery, Bambino Gesù Children’s Hospital, 00100 Rome, Italy; luigino.santecchia@opbg.net; 4 Neuromed IRCCS, 86077 Pozzili, Italy; 5 Department of Intensive Neurorehabilitation and Robotics, Bambino Gesù Children’s Hospital, 00100 Rome, Italy; maurizio.petrarca@opbg.net (M.P.); enrico.castelli@opbg.net (E.C.); 6 Sapienza University of Rome, 00185 Rome, Italy; francesca.panucciovg@gmail.com

**Keywords:** hand injuries, mirror neurons, mirror therapy, motor imagery, rehabilitation, systematic review

## Abstract

Background: Hand trauma requires specific rehabilitation protocol depending on the different structures involved. According to type of surgical intervention, and for monitoring pain and edema, post-operative rehabilitation of a hand that has experienced trauma involves different timings for immobilization. Several protocols have been used to reduce immobilization time, and various techniques and methods are adopted, depending on the structures involved. Objective: To measure the effects of mirror neurons-based rehabilitation techniques in hand injuries throughout a systematic review and meta-analysis. Methods: The protocol was accepted in PROSPERO database. A literature search was conducted in Cinahl, Scopus, Medline, PEDro, OTseeker. Two authors independently identified eligible studies, based on predefined inclusion criteria, and extracted the data. RCT quality was assessed using the JADAD scale. Results: Seventy-nine suitable studies were screened, and only eleven were included for qualitative synthesis, while four studies were selected for quantitative analysis. Four studies were case reports/series, and seven were RCTs. Nine investigate the effect of Mirror Therapy and two the effect of Motor Imagery. Quantitative analyses revealed Mirror Therapy as effective for hand function recovery (mean difference = −14.80 95% Confidence Interval (CI) = −17.22, −12.38) (*p* < 0.00001) in the short term, as well as in long follow-up groups (mean difference = −13.11 95% Confidence Interval (CI) = −17.53, −8.69) (*p* < 0.00001). Clinical, but not statistical, efficacy was found for manual dexterity (*p* = 0.15), while no benefit was reported for range of motion. Conclusions: Mirror neurons-based rehabilitation techniques, combined with conventional occupational and physical therapy, can be a useful approach in hand trauma. Mirror therapy seems to be effective for hand function recovery, but, for motor imagery and action observation, there is not sufficient evidence to recommend its use. Further research on the efficacy of the mirror neurons-based technique in hand injury is recommended.

## 1. Introduction

In 2017, the age-standardised incidence of hand and wrist fractures was 179 per 100,000 (95% uncertainty interval (UI) 146 to 217), whereas less common injuries of thumb, and non-thumb, digit amputation were 24 (95% UI 17 to 34) and 56 (95% UI 43 to 74) per 100,000, respectively [1]. Traumatic hand injury is common, causing 6.6-28.6% of Accident and Emergency visits [2]. Complex hand traumas pose a compelling therapeutic challenge for both surgery and rehabilitation. The essence of the problem lies in the coexistence of injuries to the locomotor system (muscles, tendons, bones) and problems to other structures, such as the vascular and nervous systems— leading to a high risk of loss of limb function. The ultimate goal of hand therapy is to reach the maximum level of functioning, ensuring activity and participation. The main objectives of rehabilitation programs focus on increasing range of motion (ROM) and muscle strength. However, muscle strength and ROM alone cannot be sufficient for proper hand function [3]. To balance the protection of surgical sutures, contain pain and edema, but avoid secondary osteoarthritis, the post-operative rehabilitation of the traumatic hand must modulate different splints, different immobilization times and any new approaches [4,5]. Operative and conservative treatment of trauma and degenerative diseases of the hand rely on immobilization of the affected structures [6]. Important features of immobilization devices include accurate fit to the impaired hand and preservation of non-affected hand functions [7,8].

For the recovery of hand function, different mirror neurons-based approaches can be used, namely mirror therapy (MT), action observation (AO) and motor imagery (MI).

MI is the mental rehearsal of physical movement of a body part. MI-based neurorehabilitation can be used in all stages of stroke recovery, and it serves as a supplement to conventional rehabilitation that enhances motor function [9]. MI represents a class of exercises in which an internal representation of a movement is repeatedly simulated in a first-person perspective, without actual physical movement, and seems to be effective for motor recovery in neurological and orthopedic rehabilitation [10,11]. According to Jeannerod [12] Mental Practice and preparation for a movement share common mechanisms and are functionally equivalent [13]. Furthermore, activation of mirror neurons has intimate connections with visual processing areas, activating the primary motor cortex, which is necessary for mimicking motor action [14].

MT has been widely used as a rehabilitation technique. The mirror in the patients’ midsagittal plane can reflect the movements of patients’ unaffected limbs superimposed on the impaired limbs’ position and create the visual illusion that patients’ impaired limbs can move normally [15]. MT has been primarily studied for two different purposes: pain relief [16], and post-stroke motor recovery [17]. Additionally, MT has been shown to increase ipsilateral excitability of the primary motor cortex in healthy subjects [18]; this may explain the improvement in motor function.

AO therapy assumes that while observing someone perform everyday actions, the observer’s neural networks react as if they are performing the action [19]. AO is a novel rehabilitation strategy for both adults and children. It involves observation of meaningful actions with the intention to imitate, and then performing those actions. AO is based on neurophysiological knowledge that observation of a goal-directed action activates the same neural substrate as doing the physical execution of the observed action [20].

These mirror neurons-based rehabilitation techniques have demonstrated their effectiveness in post-stroke rehabilitation [17,21], and in pain symptomatology [16]. There are still few studies in which these techniques are applied in the rehabilitation of hand injuries, but some evidence is now emerging. Therefore, the objective of the present investigation is to measure the efficacy of different mirror neurons-based rehabilitation techniques in the recovery of hand function after a hand injury through systematic review and meta-analysis.

## 2. Methods

This study was conducted by a research group composed of rehabilitation professionals and medical doctors from the Bambino Gesù Children Hospital, Sapienza University of Rome, and the ‘Rehabilitation & Outcome Measure Assessment’ (ROMA) association, who collaborate in different studies for hand therapy and systematic reviews [22,23,24,25,26,27].

### 2.1. Protocol and Registration

The Review protocol was approved into Prospero database (CRD42021240385). This review was carried out following the 27-item Preferred Reporting Items for Systematic Reviews and Meta-Analyses (PRISMA) Statement for Reporting Systematic Reviews checklist [28,29].

### 2.2. Search Strategies and Selection of the Studies

A systematic literature review was undertaken by looking for studies that investigate the effect of mirror neurons-based rehabilitation techniques in hand injuries. Search strategies were conducted from the inception to 26 February 2021. Considering the novelty of these approaches, no limitations were applied for study design, neither for the language of publication. If there was an article written in a language in which the working group was not proficient, the authors consulted official translators working in the healthcare sector. To obtain a comprehensive landscape of literature review, all studies using MT, AO, MI as sole approach and/or combined with conventional physical therapy and/or occupational therapy were included. The research focused on the adult population (>18 years) who suffered hand injuries, intended as general or unspecified injuries to the hand (according to MeSH Term); so, people with wrist/hand/finger bone fractures, tendon/peripheral nerve lesions, amputations, burns were considered for the present investigation. No restrictions were applied to the publication period, or to the country in which the study was conducted. Papers with a population showing psychiatric disorders, or studies investigating the efficacy of mirror neurons techniques in central nervous system diseases, were excluded.

The research was carried out on five electronic databases: Cinahl, Scopus, Medline, PEDro, OTseeker. The databases were queried from inception until 8 February 2020. The following electronic search strategies were used: (Hand Injuries [MeSH Major Topic]) AND Mirror Therapy; (Hand Injuries [MeSH Major Topic]) AND Motor Imagery; (Hand Injuries [MeSH Major Topic]) AND Action Observation. The search was adapted to different databases, as needed. No filters were used to avoid the loss of potentially interesting documents.

All potential papers were subjected to the screening process and the principal researcher’s analysis, and the second operator carried out the double-check. Before starting the review process, we filtered duplicate documents with Excel. Following the PRISMA checklist guidelines [29] we first screened titles, keywords and abstracts independently. After the first screening, reviewer 1 selected relevant documents, according to inclusion and exclusion criteria. Then, a second reviewer crosschecked the studies. After the second screening, studies that did not fit the inclusion criteria were excluded. A final list of studies that were eligible for inclusion was compiled, and any disagreements were resolved by a third reviewer, or by consensus. The studies that met the criteria were reviewed in the full text to determine whether they should be included in the review.

### 2.3. Data Extraction and Quality Assessment

We used special tables in which the following main data was concerned: type of study, mirror neuron-based technique, sample, interventions, outcome, follow-up, results.

The studies’ content and methodology were analyzed qualitatively, summarizing the main finding according to the use of a mirror neuron-based rehabilitation technique. For quality evaluation of the RCTs included in the meta-analysis, the Jadad scale was used to give each article a score between 0 and 5 points. Articles with a score 0–2 were considered as being of poor quality, while articles with a score equal to, or greater than, 3 were considered good quality.

### 2.4. Statistical Analyses

Review Manager software (RevMan, the Cochrane Collaboration, London, UK) was used to perform a meta-analysis. The mean difference (MD) was used as the effect size for continuous outcomes. Considering the heterogeneity of the study population, a random-effect model was used, as we expected a random effect size from the studies. The overall effect sizes were calculated based on the pooled proportions and 95% confidence intervals (CIs). The differences between the studies were calculated through the overall effect size (Z), with a statistical significance threshold of *p* < 0.05.

## 3. Results

### 3.1. Search Results

**Figure 1:**
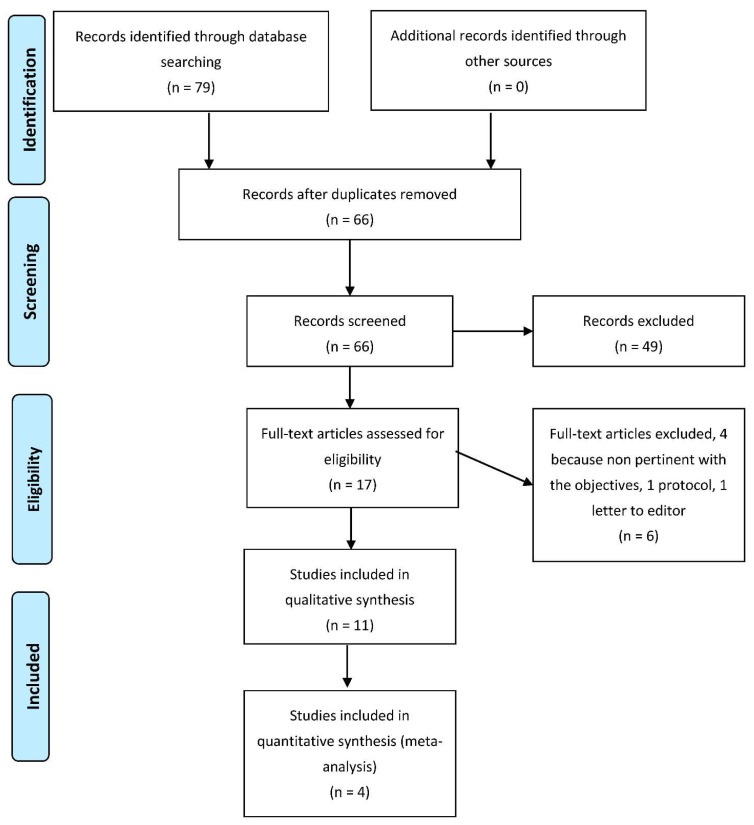
Flow-chart of search and screening process.

Figure 1 shows the selection process of the study. The total number of articles retrieved from databases was 79. Once duplicates were removed (13 research papers), we analyzed the full text of 66 articles; only 11 were included for qualitative synthesis, and 4 for quantitative analysis.

### 3.2. Characteristics of the Included Studies

The eleven studies were published between 2005 and 2019. Four studies were case reports [30,31,32,33], and seven were RCTs [34,35,36,37,38,39,40]. All studies investigated the effect of different mirror neurons-based rehabilitation techniques in adults. In particular, nine investigated the effect of MT and two the effect of MI. No studies investigated the effect of AO. Detailed information on the included studies is reported in Table 1.

The four case-report/series described the effect of mirror therapy in different hand traumas, namely brachial plexus avulsion, peripheral nerve, and orthopedic injuries. The main interventions focused on pain and sensation, while only a case report investigated the effect of MT on ROM recovery. Timing of intervention was usually with an average time of 15 min for MT combined with occupational and/or physical therapy. Authors affirm that MT can contribute to reducing pain and increases sensation and active ROM.

### 3.3. Motor Imagery

Only one RCT [34] investigated the effect of MI in burn patients. A total of 14 people with a burned hand were included in the study, and randomly allocated to the experimental group (n 9) and control group (n 5). Five sessions of MI were applied during the first two weeks of the total five weeks of rehabilitation. The authors reported that MI contributed to better motor recovery in terms of ROM. Pain outcome, as measured with VAS, was finally not reported, considering the heterogeneity of the sample and conditions.

One RCT investigated the effect of motor imagery in flexor tendon injuries [40]. The experimental group (12 people) performed MI during the immobilization phase, then followed a specific protocol for rehabilitation, as did the control group as well (13 people). The results reported that MI positively influenced central aspects of hand function, while for ROM and hand function MI seemed not to contribute to any clinical improvement.

### 3.4. Mirror Therapy

Four studies [35,37,38,39] investigated the effect of MT in people with tendon and/or nerve injuries of the hand. Sample size ranges were from 11 to 40 people. For each study, the duration of the MT session was 30 min, 2 to 5 times a week for 3 to 11 weeks, combined with conventional occupational and/or physical therapy. Primary outcomes investigated pain, sensation, and hand functions. TheRostami study [38] found that MT led to improvements for both ROM and hand function in post-intervention; while, after 3 weeks follow-up, only improvement in hand function was registered. Hand function recovery, as measured with DASH, is also reported in Abolfazi and colleagues’ study [39], while no significant differences were found in Paula’s study [35]. Hsu and colleagues’ study reported that MT revealed good effects on manual dexterity [37].

One RCT reported the effect of MT in people with mutilating injuries [36]. The experimental group (15 people with a mean age 54.8 ± 10.73) performed conventional physical therapy combined with MT (30 min daily, 3 days a week for 4 weeks). The authors investigated the effect on muscle elasticity, pain and hand function. Results reported efficacy for both outcomes in post-intervention. No follow-up was reported.

### 3.5. Trial Quality and Risk of Bias

The scoring with the Jadad scale revealed 3 RCTs of poor quality, while four obtained a score equal to, or greater than, 3 and were evaluated as good quality (see Table 1). The main problems with the articles receiving a low score, according to the Jadad scale and Cochrane collaboration risk of bias, were the impossibility of applying a double-blind study, due to the nature of treatment, inadequate description of dropout, and withdraws and inadequate randomization method. The quality assessments were initially completed by a single reviewer and then checked for accuracy by one other reviewer.

### 3.6. Meta-Analysis of Primary Outcomes

The quantitative analysis was carried out by comparing outcomes and follow-ups. This pool was based on comparable outcomes, and comparable times of follow-up allowed consideration of four studies using MT. In particular, hand function measures with DASH, hand dexterity measures with MMDT, and ROM were investigated.

**Figure 2:**
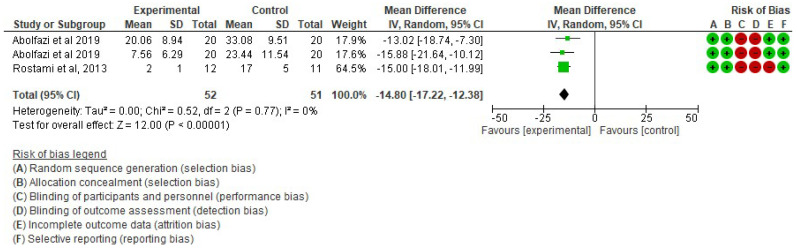
Effect of Mirror Therapy as Measured with DASH: 6–9 weeks post-intervention [38,39].

**Figure 3:**
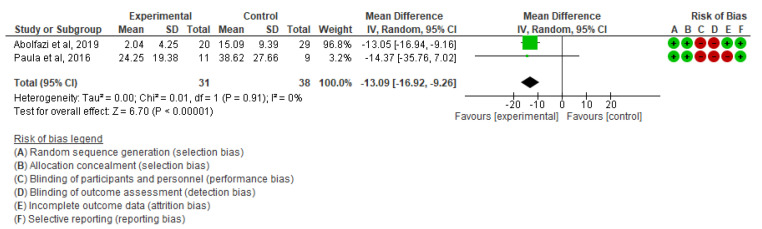
Effect of Mirror Therapy as Measured with DASH: 10–12 weeks post-intervention [35,39].

**Figure 4:**
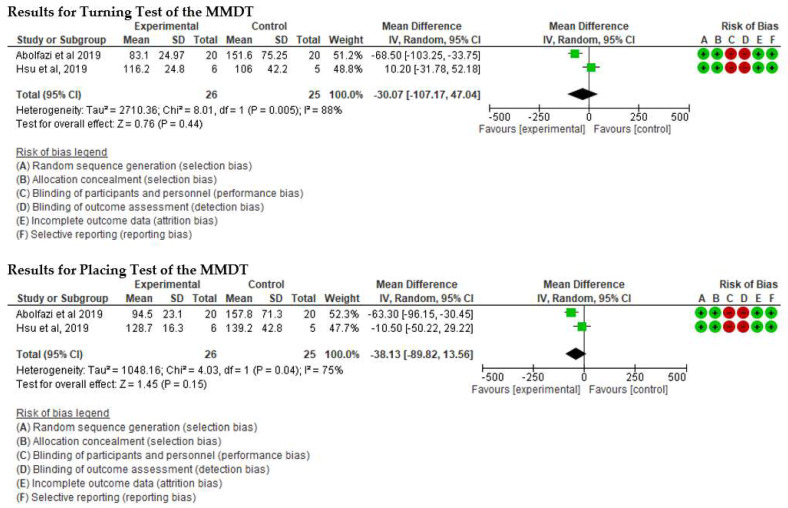
Effect of Mirror Therapy as Measured with MMDT [37,39].

**Figure 5:**
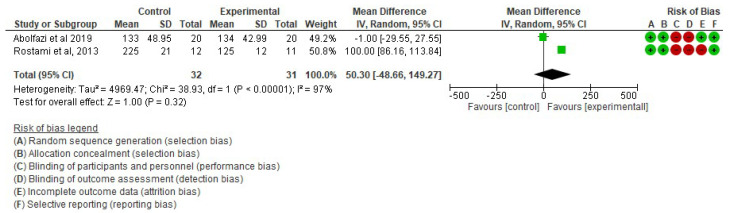
Effect of Mirror Therapy on Range of Motion after 6 weeks post-intervention [38,39].

▪ Effect of Mirror Therapy as Measured with DASH: 6–9 weeks post-intervention. The studies of Abolfazli, 2019 and Rostami, 2013 were considered. Meta-analysis revealed statistically significant results (*p* < 0.00001) in favor of the experimental group compared to the control group (mean difference = −14.80 95% Confidence Interval (CI) = −17.22, −12.38) (see Figure 2)
▪ Effect of Mirror Therapy as Measured with DASH: 10–12 weeks post-intervention. The studies of Abolfazli, 2019 and Paula, 2019 were considered. Meta-analysis revealed statistically significant results (*p* < 0.00001) in favor of the experimental group compared to the control group (mean difference = −13.11 95% Confidence Interval (CI) = −17.53, −8.69) (see Figure 3).
▪ Effect of Mirror Therapy as Measured with MMDT. The studies of Abolfazli, 2019 and Hsu, 2019. For the Turning Test of the MMDT, the meta-analysis did not reveal statistically significant results (*p* = 0.44) (mean difference = −30.07 95% Confidence Interval (CI) = −107.17, 47.04). For the Placing Test of the MMDT, meta-analysis did not reveal statistically significant results (*p* = 0.15) (mean difference = −38.13 95% Confidence Interval (CI) = −89.82, 13.56). However, for both subscales of the MMDT clinical improvement for manual dexterity was found (see Figure 4).
▪ Effect of Mirror Therapy on Range of Motion after 6 weeks post-intervention. The studies of Abolfazli, 2019 and Rostami, 2013 were considered. Meta-analysis did not reveal statistically significant results (*p* = 0.32) (mean difference = 50.30 95% Confidence Interval (CI) = −48.66, 149.27) (see Figure 5).

## 4. Discussion

In recent years, studies have shown that non-use of a limb for a short period (10–12 h) induces changes at the cortical level [41,42,43] and affects motor performance [43,44,45]. These alterations are due to immobilization causing dysfunction of proprioceptive signals to the sensory-motor system. These findings are in line with Toussaint [46] and Meugnot [47] regarding the functional plasticity of the sense-motor representation induced by a short period of immobilization (24 and 48 h) or by a sensory deprivation condition in healthy subjects [48]. The internal representation of the upper limb is therefore affected by the sense-motor deprivation caused by immobilization; this probably contributes to reducing motor performance [49].

Mental practice techniques, such as motor imagery, are commonly used to improve sports performance [49,50,51,52]. This technique is based on the concept of Central Representation Theory [53], whereby motor imagination activates the same cognitive processes as motor execution. Substantial differences, however, have been found between two motor imagery techniques; in particular, different studies have shown that KinMIP, Kinesthetic Motor Imagery Practice, has a greater association with the motor-sensory system than VisMIP, Visual Motor Imagery Practice, techniques; which, instead, mainly activate visual associative areas [34,54,55,56]. Kinesthetic motor imagery (KMI) is proprioceptive (or somato-) sensory imagination and Visual motor imagery (VMI) represents a visualization of the corresponding movement incorporating the visual network [57]. Therefore, several authors recommend the use of KinMIP for the reactivation of motor functions within rehabilitation programs [47,56,58,59], while VisMIP techniques could help build a better body schema image than KinMIP [47]. The selected studies within the present systematic review [34,40] provided preliminary evidence supporting the use of MI together with conventional rehabilitation programs. However, the findings are heterogeneous and further studies should investigate the impact of MI for hand injuries.

According to other studies selected [35,36,37,38,39], Mirror Therapy plays a role in hand function recovery. The studies are very heterogeneous, and this makes it difficult to compare protocols and, consequently, results. Nevertheless, Mirror Therapy has been shown to generate electroencephalographic activity in the motor cortex of the hand reflected in the mirror [60], and transcranial magnetic stimulation studies have revealed that it may additionally increase excitatory functions in the primary motor cortex [18,61]. These mechanisms could be the reasons for recovery of ROM and upper limb function, among others. Furthermore, in recent years, Mirror Therapy has been used in numerous studies; the main applications are focused on reducing pain symptomatology, such as in phantom limb syndrome in amputee patients [62,63], brachial plexus avulsions [64] and in complex regional pain syndrome (CRPS) [16,32,65] In traumatic hand pain there is a constant that must be strongly considered. A continuous and lasting nociceptive stimulus produces reorganization at the level of the primary motor and sensory cortex; the ability to recover and reorganize is closely related to the intensity of the perceived pain [66]. Therefore, Mirror Therapy, by creating an illusion of movement in a hypomobile and painful hand, creates a mismatch between proprioceptive and visual feedback and motor acts. This incongruity seems to be at the basis of the function of Mirror Therapy regarding painful symptoms. Moreover, as far as sensitivity is concerned, the mechanism at the basis of sensory recovery seems to be the same: the illusion of “being touched” produced by the mirror, seems to activate the somatosensory neurons that are activated when the hand is actually touched [67], and also contributes to maintenance, at the cortical level, of a “trace” of the area occupied by the hand; thus, facilitating recovery at the time of reinnervation [31].

The results of the present meta-analysis seem to suggest a possible advantage in using MT together with conventional physical and/or occupational therapy. In fact, short sessions of mirror therapy with specific rehabilitation protocols seem to have positive effects on hand function and dexterity. In particular, three studies with sample limits [29,32,33] revealed positive effects on hand function, as measured with DASH. The results of the selected studies suggest the integration of conventional therapy with mirror therapy techniques, as they can counteract the negative effects of post-operative immobilization and improve hand function. In particular, quantitative analysis confirms a positive impact on hand functioning. However, despite both short- and long-term follow-up, I^2^ revealed no heterogeneity (I^2^ 0%). It is important to state that for meta-analysis with few studies, heterogeneity can be biased [68]. Therefore, our findings should be interpreted with caution. Two studies [37,39] using MT in addition to conventional hand therapy, revealed clinically encouraging results for manual dexterity, but these findings should be further investigated with other studies having larger samples. Furthermore, these studies revealed high heterogeneity and, consequently, further studies are recommended. In the end, the quantitative analysis did not reveal any indications about the benefit of mirror therapy for ROM.

## 5. Limitations

The limitations of this study concern several factors. First, the reduced number of randomized clinical trials found in the literature have a low number of participants and high heterogeneity. Therefore, there is no possibility to have robust evidence. Second, although some studies investigated the same outcomes, they were measured with different tools, making comparison and the reaching of robust conclusions impossible. Third, we did not include all databases for our search strategy. Another limitation is the presence of numerous case reports and case series, which do not represent significant and relevant studies; however, they provided useful information for setting up rehabilitation intervention.

## 6. Conclusions

In conclusion, the present systematic review suggests that exploring different mirror-neurons based techniques is a possible approach for recovery after hand trauma. In particular, MT techniques can improve hand function and dexterity and should be used in different rehabilitation protocols. In the end, considering the non-invasive nature of the intervention, it could be useful to investigate this approach for children, following surgical intervention for congenital or traumatic hand disorders. Further studies should investigate these aspects.

## Figures and Tables

**Table 1:** Data Extraction of Selected Studies.

Author	Study	Technique	Sample	Interventions	Outcome	Follow-Up	Results	Jadad
Tsao J.W. et al., 2016 [30]	Case Report	MT	1 single case brachial plexus avulsion	15 min daily, 5 days/week	Pain and Sensation	1 month 8 months	MT coupled with nerve grafting may relieve phantom limb pain and restore sensation	/
Rosen B. et al., 2005 [31]	Case Series	MT	3 cases following hand surgery	Different timing, no more specified	Pain, ROM, Sensation	Different timing, no more specified	MT can contribute to restore sensation, pain and ROM after surgery of the hand	/
Selles R.W. et al., 2008 [32]	Case Series	MT	2 cases with peripheral nerve Injury (neuroma)	3–5 times each day for 15 min	Pain (VAS)	Different timing	MT can contribute to reducing pain in people with neuroma	/
Altschuler E.L. et al., 2008 [33]	Case Report	MT	1 case with fractured wrist	2–3 time each week for 15 min of MT combined with electrical stimulation	Active ROM	After treatment 3 months	MT combined with other approaches can contribute to recovery active motion	/
Guillot A. et al., 2009 [34]	RCT	MI	14 people with hand burns 9 EG, 5 CG	EG: five MI sessions combined with conventional therapy CG: conventional therapy	ROM Pain (VAS)	After 2-week period treatment	MI contributes to a better motor recovery in term of ROM. Pain outcome were not reported caused heterogeneity of medication and timing	1/5
Paula M.H. et al., 2016 [35]	RCT	MT	20 people with peripheral nerve and tendons injuries 11 EG, 9 CG	EG: Duran Protocol for tendons combined with 30 min MT CG: Duran Protocol combined with classic sensory re-education	Sensibility (Rosen Score, SWS) Function (DASH)	After 3 and 6 months	MT does not contribute to better outcome. None statistical significant differences were observed.	3/5
Yun D. et al. 2019 [36]	RCT	MT	30 people with mutilating injuries 15 EG, 15 CG	EG: conventional physical therapy combined with MT (30 min daily, 3 days a week for 4 weeks) CG: conventional physical therapy	Muscle Elasticity (MytonPRO) Pain (VAS) Function (PRWE)	None	MT combined with conventional physical therapy improves hand function and reduces pain	3/5
Hsu H. et al., 2019 [37]	RCT	MT	11 people with peripheral nerve injuries 6 EG, 5 CG	EG: touch-observation and task-based mirror therapy for 12 weeks CG: classic sensory re-education combined with 40 min hand/physical therapy	Sensibility (SWS test, Static 2 point discrimination) Dexterity (PPT, MMDT, Pinch-holding-up activity test)	After 3 months	Touch-observed and task-based mirror therapy result in improvement of sensation and manual dexterity	3/5
Rostami H.R. et al., 2013 [38]	RCT	MT	23 people with orthopedic injuries 12 EG, 11 CG	EG: hand therapy combined (30 min) with MT 30 min daily, 5 days a week for 3 weeks CG: hand therapy 30 min with other 30 min of functional tasks observing affected hand	ROM Function (DASH)	After 3 weeks	Mirror therapy contribute to better outcomes for both ROM and hand function in post-intervention. After 3 weeks improvement in hand function remain, while not significant improvement in ROM was observed.	3/5
Abolfazi M. et al., 2019 [39]	RCT	MT	40 people with different hand injuries (nerves, tendons soft tissue) 20 EG, 20 CG	EG: 30 min mirror therapy plus 45 conventional rehabilitation twice a week for 8 weeks CG: 75 min conventional rehabilitation	ROM Pain (McGill) Function (DASH) Strenght (Dynamometer) Dexterity (MMDT)	After 12 weeks	Mirror therapy combined with conventional hand therapy contribute in reducing pain and disability, and improving hand function and ROM in both short term and follow-up. This approach seems does not influence strength and grip.	1/5
Stenekes M.W. et al., 2009 [40]	RCT	MI	25 people with flexor tendons injuries 12 EG, 13 CG	EG: motor imagery during immobilization combined with protocol for tendons rehabilitation CG: protocol for tendon rehabilitation	Kinematic analysis Pain (VAS) Function (MHQ) ROM (Range of Motion Kit) Grip strength and pinch strength (digital dynamometer)	After 12 weeks	Motor imagery positively influences central aspects of hand function (ie, preparation time) during the rehabilitation after flexor tendon repair, while other hand function modalities appear to be unaffected	2/5

RCT: Randomized Control Trial; MI: Motor Imagery; MT: Mirror Therapy; EG: Experimental Group; CG: Control Group; ROM: Range of Motion; VAS: Visual Analog Scale; SWS: Semmes-Weinstein monofilament test; DASH: Disability of the Arm Shoulder and Hand; PRWE: PPT: Purdue Pegboard Test; MMDT: Minnesota Manual Dexterity Test; MHQ: Michigan Hand Outcomes Questionnaire.

## Data Availability

Not applicable.
